# Evaluation of Dental Health in Terminally Ill Patients

**DOI:** 10.25122/jml-2020-0023

**Published:** 2020

**Authors:** Minti Kumari, Tanoj Kumar, Shweta Rai, Anurag Rai, Rafat Sultana, Leena Priya

**Affiliations:** 1. Department of Public Health Dentistry, Patna Dental College and Hospital, Patna, India; 2. Department of Oral Pathology and Microbiology, Patna Dental College and Hospital, Patna, India; 3. Department of Oral and Maxillofacial Surgery, Buddha Institute of Dental Sciences and Hospital, Kankarbagh, Patna, India; 4. Department of Orthodontics, Patna Dental College and Hospital, Bankipore, Patna, India; 5. Lifeline Dental Care, Gaya, Patna, India; 6. Department of Oral Medicine And Radiology, Buddha Institute of Dental Science and Hospital, Patna, India

**Keywords:** Acromegaly, chronic obstructive pulmonary disease, Cushing’s syndrome

## Abstract

Dental health plays an imperative role in the general health and well-being of an individual. Terminally ill patients due to a compromised immune response are susceptible to a wide array of oral complications, which may affect their ability to speak and chew, leading to malnutrition.

The present study was conducted to evaluate dental health and various oral manifestations in terminally ill patients.

One hundred twenty terminally ill patients hospitalized with diseases of the respiratory tract, gastrointestinal tract, circulatory system, liver, and endocrinal disorders were included in the study. The evaluation of oral manifestations and their prevalence was done by a single examiner. The oral health was evaluated according to symptoms exhibited by the patients and clinical presentation.

Of the patients included in the study, 78 were male, and 42 were female. All the individuals were adults between 25 to 55 years of age. Out of 120 admitted terminally ill patients, 27 subjects had respiratory diseases, 17 had gastrointestinal disorders, 5 had disorders of the circulatory system, 39 had liver disorders, and 32 had endocrine disorders.

A need for added comprehension is mandatory to link the inter-relationships between dentistry and medicine to further perk up the management of the overall health of patients, which will further reinforce the partnership between dental and medical communities.

## Introduction

The oral cavity plays an essential role in many critical physiologic processes such as digestion, speech, and respiration. The oral cavity of the human body is a clear reflection of the comprehensive condition of heterogeneous tissues in human anatomy [1]. Thus, it is considered a window to the body because oral manifestations accompany many systemic diseases [2]. The oral cavity is a mirror of health or disease, a sentinel, or forewarning. It often presents signs and symptoms of systemic diseases that are frequently early and might be pathognomonic of that disease [3].

Oral health and general health are inter-reliant, influencing each other through biological, psychological, emotional, and developmental factors. Terminally ill patients are dependent on the care providers. As a body portal, it serves as a constant volley of invaders like bacteria, viruses, fungi, parasites [4]. The oral manifestations can reflect systemic diseases that may develop anywhere, varying from the oral mucosa, tongue, gingivae, bone, salivary glands and associated structures [5]. Due to a compromised immune response, terminally ill patients are susceptible to a wide array of oral complications, which may affect their ability to speak and chew, leading to malnutrition.

Maintaining oral health in terminally ill patients is crucial regarding the risk of nosocomial infections and improving patient comfort and discharge outcomes. These patients are at significant risk for poor oral health as they are elderly, malnourished, dehydrated, immunosuppressed, with a habit of smoking or alcohol history, are intubated or on high-flow oxygen, and are unable to have the dental plaque removed mechanically [6].

Palliative therapy is rendered to the patients with active, progressive, or advanced disease, with compromised oral health because of the disease itself or its treatment, focusing on their quality of life. The goals of palliative care patients include alleviating pain, infection, and dryness, as well as oral cleanliness through the elimination of dental plaque, calculus and food debris [7]. According to the fact sheet no. 217 form the World Health Organization (WHO), the highest fraction of patients receiving palliative care (38.5%) are those with cardiovascular diseases, the second-highest population (34%) have cancer, 10% have chronic respiratory diseases, 6% have acquired immune deficiency syndrome (AIDS), and 5% have diabetes [8].

Regarding respiratory tract diseases, the oral cavity is contiguous with the trachea, thus serves as a potential reservoir for the respiratory pathogens. Conditions in which the barriers are affected, like trauma, which destroy the physical barriers, hypoxia which destroys the electrical barrier, and immune suppressive conditions which affect the immunological barrier, tend to disturb the equilibrium and paves the way for the bacterial entry into the bloodstream [9].

Chronic obstructive pulmonary disease (COPD) is defined as a disease state characterized by airflow obstruction associated with gradual lung function loss. Leuckfeld et al. described the presence of the following manifestations in patients with COPD: chronic marginal periodontitis, oral thrush, dental plaque, gingival bleeding, and tooth loss [10]. Wang et al. depicted the presence of tooth loss in these patients; denture wearers have a greater amount of plaque acting as a reservoir of pathogens for the upper and lower airways. Also, these patients have a higher risk of oral cancer, tongue cancer, leukoplakia, and erythroplakia [11].

Sarcoidosis is a systemic disease with granulomas in the lungs and multi-organ adenopathies. Associated oral lesions include localized nodules, painless ulceration of the palate, the gingival, buccal and labial mucosae. A rare entity, Heerfordt-Waldenström syndrome characterized by systemic sarcoidosis, xerostomia, parotid gland swelling, uveitis, and facial nerve palsy, has been reported [12].

Pulmonary carcinoma presents with metastases of jawbones in more than 25% cases, with the mandible being more commonly affected than the maxilla, especially in males older than 50 years. The clinical presentation includes insidious paresthesia of the lower lip on the affected side, rapidly progressing local swelling and pain due to invasion of the inferior alveolar nerve, bone, and soft tissues [13].

Pulmonary tuberculosis can lead to oral lesions in both primary and secondary stages. Primary lesions are rare and present as a single, painless ulcer with regional lymphadenopathy. The secondary lesions are common, and usually present as single, indurated, irregular, painful ulcer covered by inflammatory exudates. The tongue is the most commonly affected site followed by the palate, lips, buccal mucosa, gingivae, palatine tonsil, and floor of the mouth. Salivary glands, tonsils, and uvula are also frequently involved [14].

Cystic fibrosis is a genetic disorder affecting mostly the respiratory tract, with chronic cough, dyspnea and recurrent infections. Associated oral manifestations include enlargement of the salivary glands, mainly the sublingual glands, followed by submandibular glands, due to the presence of mucous acinar cells and xerostomia, cheilosis from vitamin deficiency, tooth discoloration, and hypoplastic defects of the permanent dentition, the latter being associated with tetracycline use during the period of tooth formation [15].

Of the circulatory diseases, leukemia is a malignancy of the hematopoietic cells characterized by the proliferation of malignant leukocytes and the destruction of the bone marrow. Oral manifestations of leukemia may include oral ulcerations, gingival hyperplasia, petechial hemorrhage of the lips, palate, and tongue [16].

Gastrointestinal tract diseases such as Crohn’s disease and ulcerative colitis are subtypes of inflammatory bowel diseases. Crohn’s disease is characterized by chronic inflammation and non-caseating granulomas in the distal ileum and colon. Specific oral lesions include labial swelling and fissuring, mucosal tags, cobblestoning, mucogingivitis. Non-specific oral lesions comprise recurrent aphthous stomatitis, pyostomatitis vegetans, submandibular lymphadenopathy, halitosis, candidiasis, glossitis, mucosal discoloration, lichen planus, dysphagia, and dysgeusia. Ulcerative colitis is characterized by inflammation in the rectum and colon. The most commonly associated oral manifestation is pyostomatitis vegetans characterized by the formation of intra- and subepithelial abscesses of white-yellowish content with an erythematous and edematous base. These lesions may coalesce, giving a snail track appearance. These are commonly found on the tongue, lips, gingiva, tonsillar pillars, buccal mucosa, palate [17]. Other non-specific manifestations of the condition include aphthous ulcers, lichenoid lesion, halitosis, dysgeusia, dry mouth, coated tongue, gingivitis, and periodontitis [18].

Celiac disease is an autoimmune disease associated with damage in the small intestine villi. Patients with celiac disease show enamel hypoplasia, atrophic glossitis, glossodynia, decreased salivary flow rates, high caries index, and recurrent aphthous-like lesions [19].

Common liver diseases associated with increased morbidity and mortality include hepatitis C infection, alcoholic liver disease, liver cirrhosis, and hepatocellular carcinoma. Hepatitis C infection is accountable for 8000 to 10,000 deaths annually, with an estimated global prevalence of 2.2%, representing approximately 130 million infected individuals in the world [20]. Alcoholic liver disease is one of the ten most common causes of death in the industrialized world and is responsible for 3% of all fatalities [21, 22].

The oral cavity can reflect liver dysfunction in the form of pallor, petechiae, increased vulnerability to bruising, gingivitis, gingival bleeding, foetor hepaticus, which is a characteristic odor of advanced liver disease, cheilitis, smooth and atrophic tongue, xerostomia, bruxism and crusted perioral rash, chronic periodontal disease. Patients with alcoholic hepatitis present with glossitis, angular cheilitis, and gingivitis, sialadenosis [23]. The main disorders associated with hepatitis C virus (HCV) infection are xerostomia, Sjögren’s syndrome, sialadenitis, lichen planus and candidiasis development. Some patients may exhibit a triad of HCV infection, Sjögren’s syndrome and sialadenitis or salivary gland lymphoma [24].

Endocrinal disorders associated with the highest degree of mortality are diabetes mellitus, Cushing’s syndrome, and acromegaly. According to the WHO data, over 3 million people die worldwide from diabetes mellitus (DM) and its related complications every year. Cardiovascular complications and nephropathy are the most common complications attributed to mortality, more than hyperglycemia [25]. Among the oral manifestations related to DM are: dry mouth, tooth decay, periodontal disease and gingivitis, oral candidiasis, burning mouth syndrome, mucormycosis, aspergillosis, oral lichen planus, geographic tongue, and fissured tongue, delayed wound healing, and increased incidence of infection, salivary dysfunction, impaired tooth eruption, and benign parotid hypertrophy [26].

Untreated Cushing’s disease is associated with significant morbidity and mortality due to hypertension, insulin resistance, type 2 diabetes, dyslipidemia, and abdominal obesity, contributing to the adverse cardio-metabolic profile and subsequent disease burden. Short-term follow-up data suggest that cardiovascular disease confers a 4-fold increase in mortality among patients with persistent disease. Alveolar bone loss (i.e., osteoporosis of the jaw) can result from hypercortisolism and may lead to pathological fractures [27]. Acromegaly is characterized by autonomous overproduction of the growth hormone (GH) caused by a pituitary adenoma. Exposure to GH excess is associated with increased cardiovascular risk profile due to cardiac hypertrophy, diastolic dysfunction, myocardial valve insufficiency, insulin resistance, dyslipidemia, and obesity, which may contribute to increased cardiovascular and cerebrovascular mortality [27]. 

The most characteristic craniofacial features associated with acromegaly are protruded glabella and increased anterior face height. Mandibular prognathism and jaw thickening are due to the deposition of periosteal bone in response to the excess growth hormone. Other intraoral changes are spacing, malocclusion, aperthognathia, macroglossia, hypertrophy of palatal tissues, which may cause or accentuate sleep apnea, and buccal tipping of the teeth due to enlarged tongue.

The present study was conducted to evaluate the presence of various oral manifestations in terminally ill patients admitted to the Government General Hospital of Patna, Bihar, over two years. This study also presents a review of the oral manifestations in terminally ill patients with a wide array of systemic diseases.

## Material and Methods

This study was carried out at the Department of Community Dentistry and Public Health Medicine of Patna Dental College and Hospital and the Government General Hospital of Patna, Bihar, over two years, i.e., September 2017 to September 2019.

One hundred twenty terminally ill patients of either sex hospitalized with a wide variety of systemic ailments were included in the study. The systemic illness comprised diseases of the respiratory tract, gastrointestinal tract, circulatory system, liver, and the endocrine system. The evaluation of oral manifestations and their prevalence was done by a well experienced oral medicine expert deputed in the dental department of the hospital. A single examiner performed all oral evaluations. The oral manifestations were recorded according to symptoms exhibited by the patients and clinical presentation. All procedures performed in this study were in accordance with the ethical standards of the Patna Dental College And Hospital. Informed consent was obtained from all patients.

## Results

One hundred twenty patients were included in the study, out of which 78 were male, and 42 were female (Table 1). All the individuals were adults between 25 to 55 years of age. Out of the 120 terminally ill patients, 27 subjects had a respiratory illness, 17 had gastrointestinal disorders, 5 had disorders of the circulatory system, 39 had liver disorders, and 32 had disorders of the endocrine system.

**Table 1:** Gender distribution of the study subjects.

Total Subjects	Male (n%)	Female (n%)
120	78 (65%)	42 (35%)

Amongst the 27 subjects with respiratory illness, 9 had chronic obstructive pulmonary disease, 2 had sarcoidosis, 3 had pulmonary carcinoma (stage IV), 12 had tuberculosis and one patient had cystic fibrosis (Table 2). Amongst the COPD patients, 4 exhibited oral thrush, 3 had periodontitis, and the presence of both was noted in 2 patients. None of the patients presented with oral cancer, tongue cancer, leukoplakia, or erythroplakia. Clinical presentations in patients with sarcoidosis were nodules (n = 1) or ulcers (n = 1) and were mostly solitary. The tongue was the commonest affected site, followed by the lips, oral mucosa, palate, and gingiva. None of the patients with pulmonary carcinoma presented with metastasis to the jawbones (n = 0). Seven tuberculosis patients had a primary disease, whereas 5 had a secondary disease. Only 2 patients presented with a painless ulcer; one had it on the lateral border of the tongue, and the other had it on the palate. There was only one patient with cystic fibrosis and presented with generalized enamel hypoplasia (Table 3).

**Table 2:** Distribution of subjects with various systemic diseases.

Systemic disease	Type of Disorders	N	%
**Respiratory (27)**	Chronic Obstructive Pulmonary Disease	9	7.5
	Sarcoidosis	3	2.5
	Pulmonary carcinoma	3	2.5
	Tuberculosis	11	9.16
	Cystic Fibrosis	1	0.83
**Gastrointestinal (17)**	Crohn’s disease	6	5.0
	Ulcerative disease	8	0.66
	Celiac disease	3	2.5
**Circulatory (5)**	Acute myeloid leukemia	5	0.46
**Liver (39)**	Alcoholic liver disease	15	12.5
	Hepatitis C Infection	10	8.33
	Hepatocellular carcinoma	6	5.0
	Liver Cirrhosis	8	6.66
**Endocrinal (32)**	Diabetes Mellitus	18	15.0
	Cushing’s Syndrome	9	7.5
	Acromegaly	5	4.66

**Table 3:** Distribution of oral manifestations in patients with various respiratory disorders (n=27).

Disorder	Oral Manifestation	Number of subjects
**Chronic Obstructive Pulmonary Disease (n=9)**	Oral thrush	4
	Periodontitis	3
	Combination of both	2
**Sarcoidosis (n=2)**	Nodules	1
	Ulcers	1
	Heerfordt-Waldenström syndrome	0
**Pulmonary carcinoma (n=3)**	Metastasis of jaws	0
	Metastasis of soft tissues	0
**Tuberculosis (n=12)**	Solitary painless ulcer (Primary Lesion)	2
**Cystic Fibrosis (n=1)**	Enamel Hypoplasia	1

Amongst the 17 subjects with gastrointestinal disorders, 8 had ulcerative colitis, 6 had Crohn’s disease, and 3 had celiac disease. Among the Ulcerative colitis group, 6 had halitosis, 5 had dry mouth, all the patients presented with aphthous ulcers, and only one presented with pyostomatitis vegetans, appearing as an exophytic lesion on the palate. More than half the patients with Crohn’s disease (n = 6) had more than 1 type of oral lesion. The most common oral finding was mucogingivitis (n = 7), followed by mucosal tags (n = 4), deep ulceration (n= 2), cobblestoning (n = 3), and lip swelling (n = 3). Two out of 3 patients with celiac disease had enamel defects, and only one patient presented with an aphthous ulcer (Tables 4 and 5).

**Table 4:** Distribution of oral manifestations in patients with various gastrointestinal disorders (n=17).

Disorder	Oral Manifestation	Number of subjects
**Crohn’s disease (n=6)**	Labial swelling and fissuring	3
	Mucosal tags	4
	Cobblestoning	3
	Mucogingivitis	7
	Recurrent aphthous stomatitis	2
**Ulcerative colitis (n=8)**	Pyostomatitis vegetans	1
	Aphthous ulcer	8
	Lichenoid reaction	00
	Halitosis	6
	Dysguesia	00
	Dry mouth	5
**Celiac disease (n=3)**	Enamel hypoplasia	2
	Recurrent aphthous ulcers	1

**Table 5:** Distribution of oral manifestations in patients with various liver diseases (n=39).

Disorder	Oral Manifestation	Number of subjects
**Hepatitis C Infection (n=10)**	Xerostomia	0
	Sjogren’s syndrome	0
	Sialadenitis	0
	Lichen planus	5
	Candidiasis	2
**Alcoholic liver disease (n=15)**	Glossitis	00
	Angular cheilitis	8
	Gingivitis	6
	Combination of Angular cheilitis and gingivitis	1
	Sialadenosis	00
**Liver cirrhosis (n=8)**	Pallor	8
	Petechiae	00
	Gingivitis	3
	Foetor hepaticus	2
	Xerostomia	1
	Chronic periodontal disease	6
**Hepatocellular carcinoma (n=6)**	Metastatic tumor	1

Five patients had acute myeloid leukemia. Three out of 5 patients with leukemia presented with generalized gingival enlargement (Table 6). 

**Table 6:** Distribution of oral manifestations in patients with circulatory disorders (n=5).

Disorder	Oral Manifestation	Number of subjects
**Acute Myeloid Leukemia (n=5)**	Oral ulcerations	00
	Gingival hyperplasia	3
	Petechial hemorrhages	00

Among the 39 patients with liver disease, 15 had alcoholic liver disease, 10 had hepatitis C virus infection, 8 had liver cirrhosis, and 6 had hepatocellular carcinoma. Six out of 15 patients with alcoholic liver disease exhibited gingivitis, 8 exhibited angular cheilitis, and one patient had both gingivitis and angular cheilitis. Five out of 10 patients with HCV infection presented with lichen planus (n = 3 for the reticular type, n = 2 for the erosive type), and 2 patients presented with candidiasis, as shown in Tables 2 and 5. Six out of 8 patients with liver cirrhosis presented with periodontitis (approx. 5 teeth with a pocket depth of ≥6 mm). Only one patient with hepatocellular carcinoma had a metastatic tumor presenting as a gingival exophytic bleeding mass in relation to the mandible without bone involvement.

Of the 32 patients with endocrine disorders, 18 had diabetes mellitus, 9 had Cushing’s disease and 5 had acromegaly. Out of 18 patients with diabetes mellitus (controlled = 10, uncontrolled = 8), 12 patients presented with the main complaint of dry mouth, 5 had a combination of dry mouth and periodontitis, 4 had candidal growth. Eight out of 9 patients with Cushing’s syndrome presented with alveolar bone loss and periodontitis. Four out of 5 patients with acromegaly had class III malocclusion attributed to mandibular prognathism and two presented with macroglossia (Table 7).

**Table 7:** Distribution of oral manifestations in patients with endocrine disorders (n=32).

Disorder	Oral Manifestation	Number of subjects
**Diabetes Mellitus (n=18)**	Dry mouth	12
	Burning mouth syndrome	6
	Periodontal disease	9
	Combination of dry mouth and periodontal disease	5
	Impaired tooth eruption	00
	Delayed wound healing	00
	Gingivitis	00
	Candidiasis	4
**Cushing’s Syndrome (n=9)**	Alveolar bone loss	8
**Acromegaly (n=5)**	Mandibular prognathism	4
	Macroglossia	2
	Palatal tissue hypertrophy	00

## Discussion

The purpose of this study was to describe the oral manifestations in terminally ill individuals with limited life expectancy and advanced health conditions. The mouth has been called a mirror of the body. The oral cavity provides many diagnostic clues to systemic diseases and might be the first indication of a systemic condition [29].

In the present study, 120 patients with limited life expectancy exhibiting various systemic disorders were included. Out of the 120, 78 were male, and 42 were female. Twenty-seven subjects had a respiratory illness, 17 had gastrointestinal disorders, 5 had disorders of the circulatory system, 39 had liver disorders, and 32 had disorders of the endocrine system.

Nine patients presented with COPD, 4 exhibited oral thrush (44.44%) and 3 with periodontitis (33.33%) and 2 patients had both. Oral thrush, also known as oral candidiasis, is one of the most common side effects allied to the use of corticosteroid inhalers in COPD. It could be attributed to immune-suppression or increased salivary glucose after corticosteroid deposition in the oropharyngeal area. Our results were in concordance with the study conducted by Dekhuijzen et al., who found that increased risk of oral thrush was significantly associated with a high daily dose of inhalational corticosteroid [30]. COPD and chronic periodontitis are characterized by chronic neutrophilic inflammation, which mainly stems from the activity of enzymes released from the granules of neutrophils. The main factor responsible for triggering the immune system reaction is smoking [31]. In a study conducted by Bouaziz et al., 12 patients had sarcoidosis, 7 patients presented with oral involvement, 7 patients presented with nodules and 5 with ulcers. Sarcoidosis is characterized by the accumulation of epithelioid granulomas without caseation or staining for infectious agents and derangement of the tissue architecture [32]. Two out of 12 patients with tuberculosis presented with a painless ulcer, one on the lateral border of the tongue, and the other patient had it on the palate. Our findings were in concordance with the study conducted by Iype et al., who found that of all these oral lesions, the ulcerative form is the most common [33]. The most probable route of inoculation is the ingress of Mycobacterium tuberculosis in the sputum, entering the mucosal tissue through a small breach in the surface. The Mycobacterium may be carried to the oral tissues through a hematogenous route, gets deposited in the submucosa, causing proliferation and subsequent ulceration of the overlying mucosa [34]. Our patient with cystic fibrosis presented with enamel hypoplasia, which could be attributed to the recurrent courses of antibiotics, especially tetracycline. It is believed that Cystic Fibrosis Transmembrane Regulator (CFTR) function may directly affect enamel growth, and it was suggested that metabolic abuse during enamel development in the case of mutations in the CFTR gene leads to enamel defects in the postnatal period [35].

In our study, all eight patients with ulcerative colitis presented with aphthous ulcers and six with halitosis. Our findings were in concordance to the study conducted by Elahi et al., where 50 patients with ulcerative colitis were studied for oral manifestations. High statistical significance was detected in the case of oral ulceration (0.01), tongue coating (<0.001), and halitosis (<0.001) between severe ulcerative colitis patients compared with the patients in the control group [36]. The most common oral finding in patients with Crohn’s disease was mucogingivitis (n = 7), followed by mucosal tags (n = 4), deep ulceration (n = 2), cobblestoning (n = 3), and lip swelling (n = 3). Our results were contrary to the findings of Lauritano et al., who found that pyostomatitis vegetans is the most prevalent oral manifestation of inflammatory bowel diseases [37]. In our study, 2 out of 3 patients with celiac disease had enamel defects, and only one patient presented with an aphthous ulcer. Our findings were in concordance with the results of Cruz et al., who found that a higher occurrence of dental enamel defects was observed in patients with celiac disease (71.8%), predominantly in molars. Enamel hypoplasia may be associated with hypocalcemia, genetic susceptibility, or an autoimmune reaction in the enamel during odontogenesis [38].

In our study, 3 out of 5 patients with acute myeloid leukemia presented with generalized gingival enlargement. According to Stafford et al., oral manifestations in leukemia may either be associated with direct infiltration of leukemic cells or may be due to thrombocytopenia, neutropenia, or impaired granulocyte function [39].

Six out of 15 patients with alcoholic liver disease exhibited gingivitis, 8 exhibited angular cheilitis, one patient had both gingivitis and angular cheilitis. Five out of 10 patients with HCV infection presented with lichen planus (n = 3 reticular type, n = 2 erosive type), and 2 patients presented with candidiasis. Our findings were in concordance with the one conducted by Alaizari et al., who conducted a meta-analysis amongst 19 eligible studies, including 187 cases of oral lichen planus (OLP) and 2519 controls. There was a statistically significant difference in the proportion of HCV seropositivity among oral lichen planus patients compared to controls. It is believed that HCV acts locally to modify the function of the epithelial cells. However, the immune response of the host to HCV may be responsible for the development of OLP [40]. Six out of 8 patients with liver cirrhosis presented with periodontitis (approximately 5 teeth with a pocket depth greater or equal than 6 mm). Our study results were in concordance with the one conducted by Gronkjaer et al., which enrolled 145 patients with cirrhosis for the oral examination of various parameters: plaque, pocket depth, clinical attachment level, and bleeding on probing. It was found that most cirrhosis patients had significant periodontitis, the severity of which was related to lifestyle factors such as smoking, brushing and others [41]. Only one patient with hepatocellular carcinoma had a metastatic tumor presenting as a gingival exophytic bleeding mass in relation to the mandible without bone involvement. Most cases of metastatic hepatocellular carcinoma in the oral cavity involve the mandible and gingiva, presenting either as exophytic tumors or as intraosseous lesions [42].

Out of 18 patients with diabetes mellitus, 12 presented with dry mouth as the main complaint, 5 had a combination of dry mouth and periodontitis, and 4 had candidal growth. Our study results were in concordance with the systematic review led by Pintor et al., where the systematic literature searches in biomedical databases were done from 1970 until January 18th, 2016. All studies showed a higher prevalence of xerostomia in DM patients in relation to the non-DM population [43]. Xerostomia and disturbances of pH and glucose can encourage the Candida overgrowth in the oral cavity and are responsible for oral candidiasis in diabetic patients [44]. Eight out of 9 patients with Cushing’s syndrome presented with alveolar bone loss and periodontitis. Patients with Cushing’s syndrome are at increased risk of developing periodontal disease as cortisol inhibits white blood cells from migrating into the space between the gum and teeth where they can fight against local bacteria. Also, cortisol inhibits new bone formation, which contributes to osteoporosis of the jaw bone [45]. Four out of 5 patients with acromegaly had class III malocclusion attributed to mandibular prognathism and 2 presented with macroglossia. Mandibular prognathism can be attributed to appositional growth and hypertrophy of the condylar cartilage, which is aggravated by the pressure exerted by the tongue. The study conducted by Künzler et al. showed that 16 to 57% of the patients with acromegalia had class III malocclusion with an average protrusion of the mandible of 9.3 +/- 3.4mm [46].

## Conclusion

Oral manifestations may sometimes be the most preliminary sign of systemic diseases. Several systemic pathologies may summit to common oral manifestations. However, specific oral manifestations may be associated with a particular disease. A need for added comprehension is mandatory to link the inter-relationships between dentistry and medicine to further perk up the management of overall health of patients which will further reinforce the partnership between dental and medical communities.

## Conflict of Interest

The authors declare that there is no conflict of interest.
